# Case Report: A family history of peanut allergy and hereditary alpha-tryptasemia

**DOI:** 10.3389/falgy.2023.1322117

**Published:** 2024-01-23

**Authors:** Yannick Chantran, Hélène Renaudin, Michel Arock, Tamazoust Guiddir, Ariane Nemni

**Affiliations:** ^1^ Molecular Platform for the Analysis of cKIT Mutations and Other Gene Defects, Centre National de Référence des Mastocytoses, Saint-Antoine Hospital, DMU BioGeMH, AP-HP.Sorbonne University, Paris, France; ^2^ Department of Biological Immunology, Saint-Antoine Hospital, DMU BioGeMH, AP-HP.Sorbonne University, Paris, France; ^3^ Health Environmental Risk Assessment (HERA) Team, Centre of Research in Epidemiology and Statistics (CRESS), Inserm/INRAE, Faculty of Pharmacy, Université Paris Cité, Paris, France; ^4^ Children-Adult Allergology Department, Robert Ballanger Hospital, Aulnay Sous-Bois, France; ^5^ Department of Biological Haematology, Pitié-Salpétrière Hospital, DMU BioGeMH, AP-HP.Sorbonne University, Paris, France; ^6^ Pediatric Pulmonology and Allergology Unit, Bicêtre Hospital, AP-HP, Paris-Saclay University, Le Kremlin Bicêtre, France

**Keywords:** food allergy, anaphylaxis, tryptase, hereditary alpha-tryptasemia, case report

## Abstract

**Context:**

Hereditary alpha-tryptasemia (HαT) is associated with elevated basal serum tryptase (bST) and is associated with a higher risk of severe anaphylactic reactions in patients with clonal mast cell disorders or IgE-mediated Hymenoptera venom-induced anaphylaxis. The consequence of this genetic trait remains to be determined in other allergic diseases and food allergy in particular.

**Objectives:**

Here, we describe three cases of peanut allergy among siblings from a single family of four: two of them were associated with HαT, and the third one was associated with the tryptase wild-type genotype.

**Methods:**

*TPSAB1/TPSB2* genotypes were determined by digital PCR. After the case description, we provided a review of the literature regarding bST levels and tryptase genotypes in anaphylaxis, with a particular focus on food allergy.

**Results:**

Compared to the sibling with the conventional tryptase genotype, the two siblings with HαT presented a lower peanut threshold at the initial oral food challenge, higher peanut skin prick test reactivity, higher levels of specific IgE to peanut, Ara h 2, and Ara h 6, and a lower IgG4/IgE ratio after 10 years of oral immunotherapy.

**Conclusion:**

The tryptase genotype and HαT status might modify the clinical presentation and biological features of food allergy.

## Introduction

Elevated basal serum tryptase (bST) has been described as a risk factor for severe anaphylactic reactions, particularly in Hymenoptera venom anaphylaxis (1). One of the main determinants of bST levels is the tryptase genotype (2). In particular, hereditary alpha-tryptasemia (HαT) corresponds to additional copies of the TPSAB1 gene encoding α-tryptase and is associated with elevated bST levels, almost exclusively >8 µg/L (3). This genetic trait is present in about 5% of the population of Caucasian descent and is overrepresented among patients with clonal mast cell disorders including systemic mastocytosis (4). When associated with clonal mast cell disorders, HαT has been associated with a higher risk and severity of anaphylactic reactions to hymenoptera venom (4, 5). In addition, among patients with severe Hymenoptera venom anaphylaxis or idiopathic anaphylaxis, HαT is also present at a higher rate than in the general population, even in the absence of overt mast cell clonal disorder (4).

In food allergy, alpha-tryptase-positive genotypes, in general, have been associated with more severe food reactions (6). Here, we describe three cases of peanut allergy among siblings from a single family of four, two of them were associated with HαT, and the third one was associated with a conventional α-tryptase-positive genotype.

## Case description

We report three cases of peanut allergy among siblings from a family of four children. Two of them were associated with HαT. The mother reported atopy but no food allergy. The father and the remaining daughter did not report any history of allergic disease.

### Patient A

The 31-year-old brother (patient A) presented with a history of atopic dermatitis, remitting asthma, and allergic rhinitis to house dust mites, cats, and birch and grass pollens during childhood. In his childhood, he also experienced abdominal pain and vomiting after drinking soymilk and oral pruritus and abdominal pain after eating pasta containing cashew nuts. However, he can now consume bean curd, stem beans, and cashew nuts without experiencing any hypersensitivity reactions. In addition, he had reactions to peanuts during childhood.

In 2011, at age 20, he underwent an oral food challenge (OFC) to peanuts, which revealed a positive result at a threshold of 650 mg. Since then, he has been receiving peanut oral immunotherapy (OIT), currently with six peanut M&M’s 3 days per week. He once ate 15 peanuts without experiencing any hypersensitivity reactions. In 2018, peanut prick tests were positive for native roasted peanuts (7 mm wheal; histamine positive control: 4 mm). In 2015, the total serum IgE level was 124 kUI/L. In 2019, polysensitization to peanut molecular allergens was found, with a serum peanut-specific IgE level of 39.5 kU_A_/L, a serum Ara h1-specific IgE level of 23.6 kU_A_/L, a serum Ara h2-specific IgE level of 13.9 kU_A_/L, a serum Ara h3-specific IgE level of 9.36 kU_A_/L, and a serum Ara h6-specific IgE level of 16 kU_A_/L. Biological results after 10 years of oral immunotherapy are summarized in Table 1. In 2021, the serum peanut-specific IgG4/IgE ratio was 24.2, the serum Ara h2-specific IgG4/IgE ratio was 105.1, and the serum Ara h6-specific IgG4/IgE ratio was 83.7. Sensitization to soy molecular allergens was also investigated, with a serum soy-specific IgE level of 3.18 kU_A_/L, a serum Gly m4-specific IgE level of 5.91 kU_A_/L, a serum Gly m5-specific IgE level of 0.96 kU_A_/L, and a serum Gly m6-specific IgE level of 4.61 kU_A_/L. Of note, the serum lupine-specific IgE level was 1.19 kU_A_/L.

**Table 1:** Clinical and biological characteristics of patients after 10 years of peanut oral immunotherapy.

Patient	A	B	C
Peanut anaphylaxis	Yes	Yes	Yes
Initial peanut OFC threshold (mg)	650	1,000	1,500
Basal serum tryptase (µg/L)	14.3	9.5	3.9
Tryptase genotype *TPSAB1*/*TPSB2*	ααβ:ββ	ααβ:ββ	αβ:ββ
Hereditary alpha-tryptasemia	Yes	Yes	No
*KIT* D816V mutation in peripheral blood (>0.01%)	No	No	No
Peanut OIT maintenance dose (3 per week)	6	4–5	5–7
Peanut skin prick test (wheal in mm)	12	12	6
Peanut-specific IgE (kU_A_/L)	38.3	30.3	0.71
rAra h 2-specific IgE (kU_A_/L)	11.8	27.6	0.57
rAra h 6-specific IgE (kU_A_/L)	16	30.4	0.9
Peanut-specific IgG4/IgE ratio	24.2	18.4	163.0

Recently, he experienced three exercise-induced anaphylaxis-like reactions soon after peanut consumption: one with urticaria, one with asthma and urticaria, and then one after climbing stairs with abdominal pain and flush, which improved within 4 h with anti-histamine and corticosteroid treatment. Percritical serum tryptase levels at the time of reactions were not investigated.

**Figure 1:**
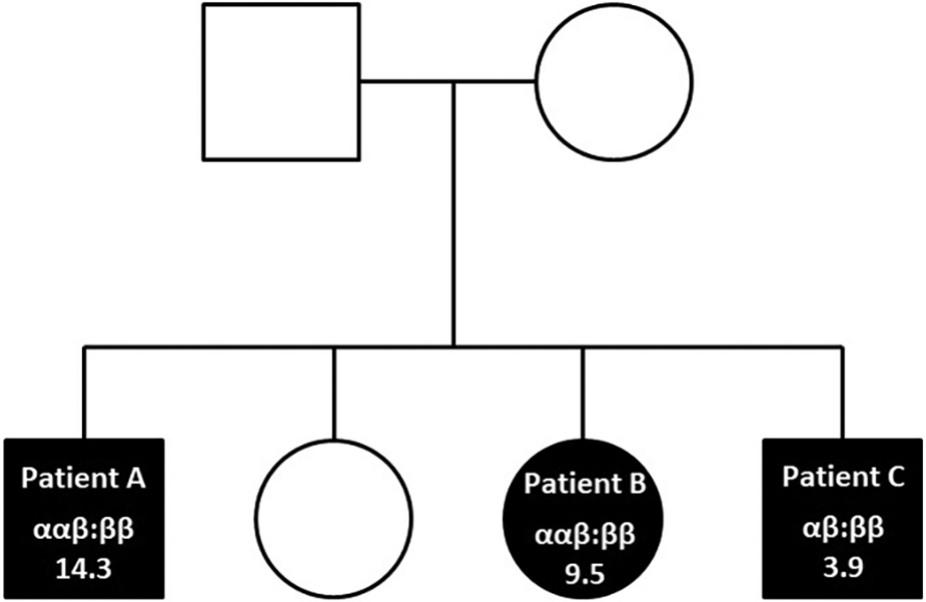
Familial pedigree showing inherited TPSAB1/TPSB2 genotypes and tryptase levels in peanut-allergic siblings (filled symbols).

The basal serum tryptase level was 14.3 µg/L. Tryptase genotyping of *TPSAB1* and *TPSB2* was performed using droplet digital PCR, as described by Lyons et al. (3). Patient A presented HαT with *TPSAB1* duplication and ααβ:ββ genotype (Figure 1). Research of *KIT* D816V mutation on peripheral blood by digital PCR was found negative.

### Patient B

The 25-year-old sister (patient B) presented with a history of atopic dermatitis, persistent asthma, and allergic rhinitis with sensitization to house dust mites, cats, and birch and grass pollens. In 2005, at 9 years old, she presented with airborne conjunctivitis, labial angioedema, and pruritus after carrying peanuts and nuts. In 2011, an oral food challenge to peanuts revealed positive results at a threshold of 1,000 mg. In April 2012, after eating half a pad Thai on top of which she had removed peanuts, she presented with labial and pharyngeal pruritus, abdominal pain, and vomiting. Since 2011, patient B has been receiving peanut oral immunotherapy with 4–5 peanut M&M's 3 days per week, as well as budesonide/formoterol 400 b.i.d., montelukast 10 mg once a day, ebastine once a day, and azelastine 1,000 µg/fluticasone propionate 365 µg b.i.d.

In 2016, peanut prick tests were positive for native roasted peanuts (8 mm wheal; histamine positive control: 3 mm). In 2019, polysensitization to several peanut molecular allergens was found, with a serum peanut-specific IgE level of 54.9 kU_A_/L, a serum Ara h1-specific IgE level of 4.73 kU_A_/L, a serum Ara h2-specific IgE level of 38 kU_A_/L, a serum Ara h3-specific IgE level of 1.84 kU_A_/L, and a serum Ara h6-specific IgE level of 41.4 kU_A_/L. In 2021, after 10 years of oral immunotherapy, the serum peanut-specific IgG4/IgE ratio was 18.4, the serum Ara h2-specific IgG4/IgE ratio was 16.6, and the serum Ara h6-specific IgG4/IgE ratio was 20.1. Of note, the serum lupine-specific IgE level was 14.4 kU_A_/L. Other biological results after 10 years of oral immunotherapy are summarized in Table 1.

The basal serum tryptase level was 9.5 µg/L. Tryptase genotyping revealed HαT with *TPSAB1* duplication and ααβ:ββ genotype (Figure 1). Research of *KIT* D816V mutation in peripheral blood was found negative.

### Patient C

The 23-year-old brother (patient C) presented with a history of remitting asthma and allergic rhinitis. After reactions to peanuts during childhood, an oral food challenge was performed in 2011 and revealed positive at a threshold of 1,500 mg. Oral immunotherapy was initiated and is still ongoing. Patient C is now receiving peanut oral immunotherapy with 5–7 peanut M&M's once a week. In 2014, after a 3-month discontinuation of a 20-mg-peanut-dose oral immunotherapy, he presented with abdominal pain after peanut exposure, which was treated with corticosteroids and phloroglucinol, and resumed oral immunotherapy. Later, he reported having eaten once 10 peanuts without allergic reactions.

In 2018, peanut prick tests were positive with a 6-mm wheal for native roasted peanuts and a 5-mm wheal for histamine control. In 2019, sensitization to serum peanut molecular allergens was evaluated, with a serum peanut-specific IgE level of 1.16 _A_/L, a serum Ara h1-specific IgE level of <0.10 kU_A_/L, a serum Ara h2-specific IgE level of 0.80 kU_A_/L, a serum Ara h3-specific IgE level of <0.10 kU/L and a serum Ara h6-specific IgE level of 1.21 kU_A_/L. Biological results after 10 years of oral immunotherapy are summarized in Table 1. The total serum IgE level was 214.6 kU/L. The serum peanut-specific IgG4/IgE ratio was 18.4, the serum Ara h2-specific IgG4/IgE ratio was 16.6, and the serum Ara h6-specific IgG4/IgE ratio was 20.1.

The basal serum tryptase level was 3.9 µg/L. Consistent with tryptase levels, no HαT was found but the conventional αβ:ββ tryptase genotype (Figure 1). No cKit D816V mutation was found in peripheral blood.

## Discussion

To the best of our knowledge, this is the first description of HαT in a family with peanut allergy. Interestingly, the two siblings with HαT presented a lower peanut threshold at the initial oral food challenge, higher peanut skin prick test reactivity, higher levels of specific IgE to peanuts, Ara h 2, and Ara h 6, and a lower IgG4/IgE ratio after 10 years of oral immunotherapy compared to the third sibling who displayed a conventional genotype.

Although limited to the description of siblings from a single pedigree, this study allows the unique comparison of HαT status and clinical or biological variates in individuals with similar parental history, overall genetic background, and personal history of anaphylaxis to the same food. All were treated with oral immunotherapy for the same duration, underwent serological evaluation at the same follow-up time point, and were evaluated by the same physician. No bone marrow studies were performed due to the absence of argument suggesting a clonal mast cell disorder, despite slightly elevated tryptase levels in two siblings, later explained by the presence of HαT. Evaluation of the *KIT* D816V variant allelic fraction in the peripheral blood was performed, but the result showed a negative finding.

Several studies have underlined the relationship between bST levels and the risk and severity of anaphylaxis in patients with Hymenoptera venom allergy (7–10), in children with food allergy (11, 12), and in adults with cofactor-dependent wheat allergy (13). Most conditions associated with higher bST levels, such as male gender, older age, cardiovascular conditions, or clonal mast cell disorders, are also risk factors for severe anaphylaxis (1, 7, 14). In the case of HαT, the vast majority of HαT^+^ patients exhibit bST levels ≥8 μg/L, with some falling in the range between 6 and 8 μg/L (15). Initial studies revealed that bST levels in HαT follow a gene–dosage effect, meaning that a higher gene copy number is associated with higher bST levels (3). However, tryptase over-expression in HαT now appears mainly related to an enlarged over-active promoter element co-inherited with additional *TPSAB1* copies (2). In exceptional cases, individuals with numerous additional *TPSAB1* copies might even exhibit bST levels above 100 μg/L (2). In clonal mast cell disorders, increased HαT prevalence was consistently reported in several cohorts compared to the general population (4, 5, 16–18). In addition, there is a strong consensus that in clonal mast cell disorders, HαT is a modifier of the frequency and severity of anaphylaxis to hymenoptera venom and likely idiopathic anaphylaxis (4, 5, 16, 17). Conversely, the prevalence of HαT appears elevated in patients with a history of grade IV Hymenoptera venom anaphylaxis or idiopathic anaphylaxis, even in the absence of clonal MC disorder (4).

It still remains unknown whether HαT is also overrepresented among patients experiencing severe drug or food anaphylaxis in the absence of clonal mast cell disorder. However, food intolerances were reported as a frequent complaint in patients with HαT, affecting up to 40% of patients with HαT referred for elevated bST (19). Moreover, in seminal papers about HαT, out of 10 anaphylaxis triggers reported in 33 patients, two were foods, two were Hymenoptera stings, and one was an idiopathic reaction (20). Similarly, in a cohort of 101 patients with HαT referred for mast cell activation-related symptoms and without clonal mast cell disorders, 57.4% presented doctor-diagnosed anaphylaxis: drugs were the most frequent trigger (52%), followed by foods (29%), venoms (17%), and idiopathic reactions (14%) (21). Recently, Lang et al. reported that not only H*α*T but all *α*-allele-bearing genotypes, including conventional αβ:ββ and αβ:αβ genotypes, were associated with a higher risk of anaphylaxis among children with food allergy compared with the ββ:ββ genotype. Children with food allergy and an α-tryptase^+^ genotype also tended to present more severe reactions. In a second cohort of children with peanut allergy, individuals with α-tryptase^+^ genotypes had higher total severity scores during oral food challenge than those with the ββ:ββ genotype. Moreover, symptom severity scores in this group positively correlated with the α-tryptase copy number (6). The specific properties of α/β-tryptase heterotetramers present in individuals expressing α-tryptase, such as EMR2 pre-activation or protease-activated receptor-2 (PAR2) activation, provide the conceptual basis for differences in allergic phenotypes according to the HαT status or even the conventional tryptase genotype (22).

In conclusion, within the context of scarce data regarding the relationship between HαT status and food allergy, this study provides more insight into the serological and clinical correlates of HαT. Siblings with HαT presented with a lower reaction threshold at the initial challenge, but after 10 years of oral immunotherapy, they displayed higher sensitization levels and lower IgG4/IgE ratios compared to the sibling with a wild-type genotype. Cohort studies are needed to confirm this association.

## Patient perspective

All three patients declared a satisfying quality of life under oral immunotherapy. Regarding the two patients with tryptase elevation, concordant tryptase genotypes and negative *KIT* D816V in peripheral blood without other manifestations of systemic mastocytosis were comforting for the patients. The uncertainties related to the HαT status in the context of food allergies were explained to the patients, as well as the reassurance about the very low risk of severe allergic reactions in their progeny.

## Data Availability

The original contributions presented in the study are included in the article/Supplementary Material, further inquiries can be directed to the corresponding author yannick.chantran@aphp.fr.
